# Laparoscopic versus open resections in the posterosuperior liver segments within an enhanced recovery programme (ORANGE Segments): study protocol for a multicentre randomised controlled trial

**DOI:** 10.1186/s13063-022-06112-3

**Published:** 2022-03-09

**Authors:** Christoph Kuemmerli, Robert S. Fichtinger, Alma Moekotte, Luca A. Aldrighetti, Somaiah Aroori, Marc G. H. Besselink, Mathieu D’Hondt, Rafael Díaz-Nieto, Bjørn Edwin, Mikhail Efanov, Giuseppe M. Ettorre, Krishna V. Menon, Aali J. Sheen, Zahir Soonawalla, Robert Sutcliffe, Roberto I. Troisi, Steven A. White, Lloyd Brandts, Gerard J. P. van Breukelen, Jasper Sijberden, Siân A. Pugh, Zina Eminton, John N. Primrose, Ronald van Dam, Mohammed Abu Hilal, on behalf of the ORANGE trials collaborative

**Affiliations:** 1 Hepatobiliary and Pancreatic Surgical Unit, University Hospital Southampton NHS Foundation Trust, Southampton, SO16 6YD UK; 2 Department of Surgery, Foundation Poliambulanza, Via Bissolati, Brescia, Italy; 3 Department of Surgery, Maastricht University Medical Centre+, 6202 AZ Maastricht, The Netherlands; 4 Department of Surgery and Transplantation, University Hospital RWTH Aachen, Aachen, Germany; 5 Hepatobiliary Surgery Division, IRCCS San Raffaele Hospital, Milan, Italy; 6 Peninsula HPB Unit, Derriford Hospital, Plymouth Hospitals NHS Trust, Plymouth, UK; 7 Department of Surgery, Amsterdam UMC, University of Amsterdam, Cancer Center Amsterdam, Amsterdam, The Netherlands; 8 Department of Digestive and Hepatobiliary/Pancreatic Surgery, Groeninge Hospital, Kortrijk, Belgium; 9 Hepatobiliary Surgery Unit, Aintree University Hospital, Liverpool, UK; 10 Department of HPB Surgery, Oslo University Hospital, Oslo, Norway; 11 Department of Hepato-Pancreato-Biliary Surgery, Moscow Clinical Research Centre, Moscow, Russia; 12 Division of General Surgery and Liver Transplantation, San Camillo Hospital, Rome, Italy; 13 Institute of Liver Studies, Kings College Hospital, London, UK; 14 Department of Surgery, Manchester University Foundation Trust, Manchester, UK; 15 Department of Surgery, Oxford University Hospital NHS Foundation Trust, Oxford, UK; 16 Department of Hepatobiliary and Pancreatic Surgery, University Hospitals Birmingham, NHS Foundation Trust, Birmingham, UK; 17 Division of HPB, Minimally Invasive and Robotic Surgery, Department of Clinical Medicine and Surgery, Federico II University Hospital, Naples, Italy; 18 Department of HPB and Transplant Surgery, Freeman Hospital, Newcastle upon Tyne, UK; 19 Clinical Epidemiology and Medical Technology Assessment (KEMTA), Maastricht UMC+, Maastricht, The Netherlands; 20 Department of Methodology and Statistics, Maastricht University Medical Centre, Maastricht, The Netherlands; 21 Department of Oncology, Addenbrooke’s Hospital, Cambridge, UK; 22 Southampton Clinical Trials Unit, University of Southampton, Southampton, UK; 23 Department of Surgery, University of Southampton, Southampton, UK; 24 GROW – School for Oncology and Developmental Biology, Maastricht University, Maastricht, The Netherlands

**Keywords:** Laparoscopy, Liver surgery, Posterosuperior segments, Randomised controlled trial, Enhanced recovery

## Abstract

**Background:**

A shift towards parenchymal-sparing liver resections in open and laparoscopic surgery emerged in the last few years. Laparoscopic liver resection is technically feasible and safe, and consensus guidelines acknowledge the laparoscopic approach in the posterosuperior segments. Lesions situated in these segments are considered the most challenging for the laparoscopic approach. The aim of this trial is to compare the postoperative time to functional recovery, complications, oncological safety, quality of life, survival and costs after laparoscopic versus open parenchymal-sparing liver resections in the posterosuperior liver segments within an enhanced recovery setting.

**Methods:**

The ORANGE Segments trial is an international multicentre randomised controlled superiority trial conducted in centres experienced in laparoscopic liver resection. Eligible patients for minor resections in the posterosuperior segments will be randomised in a 1:1 ratio to undergo laparoscopic or open resections in an enhanced recovery setting. Patients and ward personnel are blinded to the treatment allocation until postoperative day 4 using a large abdominal dressing.

The primary endpoint is time to functional recovery. Secondary endpoints include intraoperative outcomes, length of stay, resection margin, postoperative complications, 90-day mortality, time to adjuvant chemotherapy initiation, quality of life and overall survival.

Laparoscopic liver surgery of the posterosuperior segments is hypothesised to reduce time to functional recovery by 2 days in comparison with open surgery. With a power of 80% and alpha of 0.04 to adjust for interim analysis halfway the trial, a total of 250 patients are required to be randomised.

**Discussion:**

The ORANGE Segments trial is the first multicentre international randomised controlled study to compare short- and long-term surgical and oncological outcomes of laparoscopic and open resections in the posterosuperior segments within an enhanced recovery programme.

**Trial registration:**

ClinicalTrials.gov NCT03270917. Registered on September 1, 2017. Before start of inclusion. Protocol version: version 12, May 9, 2017

**Supplementary Information:**

The online version contains supplementary material available at 10.1186/s13063-022-06112-3.

## Background

The mainstay of curative treatment for both primary and secondary malignant hepatic disease is surgery [1–3]. Traditionally, an open approach has been used typically necessitating a large upper abdominal incision. Over the last two decades, there has been a progressive move towards the increased use of minimally invasive techniques [4–6]. Alongside this, there has been a shift from the traditional anatomical resections towards parenchymal-sparing liver resections. In fact, in both open and laparoscopic surgery, the parenchymal-sparing approach has become the preferred technique for the treatment of a variety of liver lesions [7–11].

The feasibility, safety and oncological effectiveness of a liver resection are determined by many factors. These include patient factors, such as underlying liver disease and previous abdominal surgery, and anatomical factors, such as the extent of resection, tumour size and location and proximity to major vessels together with the surgeon’s experience [12–16]. The location of the tumour is of particular relevance, with lesions situated in the posterosuperior segments (PSS) considered to be the most challenging. This applies to both laparoscopic and open surgery owing to the difficulty in gaining adequate access to perform a safe resection. In open surgery, a very large incision and an extensive liver mobilisation are often required, while advanced laparoscopic skills and a wide experience in laparoscopic liver surgery are needed when performed laparoscopically.

The first international consensus meeting on laparoscopic liver surgery held in Louisville in 2008 considered lesions in the PSS as a contraindication to minimally invasive surgery. The subsequent Morioka consensus conference in 2014 considered resections in this location to be technically major resections [17]. More recently, the Southampton consensus guidelines in 2017 acknowledged that laparoscopic resections for lesions in the PSS are feasible and safe and should be considered as a valid alternative approach in expert centres [18]. This was supported by observational studies showing an association between the laparoscopic approach and superior short-term outcomes including lower blood loss, fewer postoperative complications, lower analgesic requirements, shorter hospital stay and shorter time to commencing postoperative chemotherapy [19–25]. In addition, oncological effectiveness has been postulated to be at least comparable between the two techniques [17, 18, 26, 27].

It has been suggested that the benefits from the minimally invasive approach may be greater for resection of the posterosuperior segments, since patients are spared a very large incision for a relatively small liver resection [6, 12, 28, 29]. Minimisation of surgical trauma is also a key component of the established enhanced recovery programmes that have proven to shorten hospital stay, reduce complications and lower costs [30–35]. Both may very well complement each other in a harmonious way. It is therefore crucial to assess the benefit of a laparoscopic approach within these programmes.

## Methods

The trial protocol is written in accordance with SPIRIT guidelines (supplementary Fig. 1 and supplementary document 1) [36].

### Study aim

The ORANGE Segments trial aims to determine whether laparoscopic resection of the posterosuperior liver segments is superior to an open approach, in terms of short- and long-term surgical and oncological outcomes, when performed in an enhanced recovery setting. The hypothesis is that laparoscopic surgery will result in a shorter time to functional recovery, fewer postoperative complications and comparable oncologic outcomes, as compared to open surgery.

The objectives of this study are the following:

1. To compare the time to functional recovery after laparoscopic and open liver resection in the posterosuperior segments
2. To compare the intraoperative blood loss, operation time, intraoperative incidents (Satava classification), length of hospital stay, morbidity, liver-specific morbidity, 90-day mortality, readmission percentage, resection margin, quality of life, body image, incidence of incisional hernia at 1 year, hospital and societal costs for 1 year, time to adjuvant chemotherapy initiation, disease-free survival and overall survival

### Study design and setting

The ORANGE Segments trial is designed as an international multicentre randomised controlled trial, with patients and ward personnel blinded to the treatment intervention. All patients participate within the locally implemented enhanced recovery programme.

Preoperatively, the patients’ baseline characteristics are recorded. Intraoperative and postoperative data is documented during the admission and also following discharge at 10 days and 3, 6 and 12 months after surgery. The ORANGE Segments trial protocol is added to the protocol and the ethical permissions for the successfully completed ORANGE II PLUS trial after appropriate major amendments. This allowed for an efficient and cost-effective start-up of the trial across 16 centres and 7 countries in Europe. The monitoring is performed by the same independent Data and Safety Monitoring Board (DSMB).

All the centres involved have considerable experience in performing both open and laparoscopic liver surgery. Nonetheless, an unedited video of the surgeon from the centre performing laparoscopic surgery in the posterosuperior segments is assessed by the chief investigator (MAH) before trial entry. A minimum of 10 laparoscopic resections in the posterosuperior liver segments overall per surgeon are required.

#### Inclusion criteria

- Patients requiring a parenchymal-sparing liver resection (including wedge resections and full segmentectomies) involving one or two of segments 4a, 7, 8 or a segment 6/7 resection for accepted indications
- Men and non-pregnant, non-lactating women between age 18 years and older
- BMI between 18 and 35 kg/m^2^
- Patients with ASA physical status I, II or III
- Able to understand the nature of the study and what will be required

#### Exclusion criteria

- Inability to provide written informed consent
- Patients with hepatic lesion(s) located with insufficient margin from vascular or biliary structures to be operated laparoscopically in the opinion of the treating surgeon
- Patients with ASA physical status higher than III
- Repeat hepatectomy

### Treatment group

**Fig. 1:**
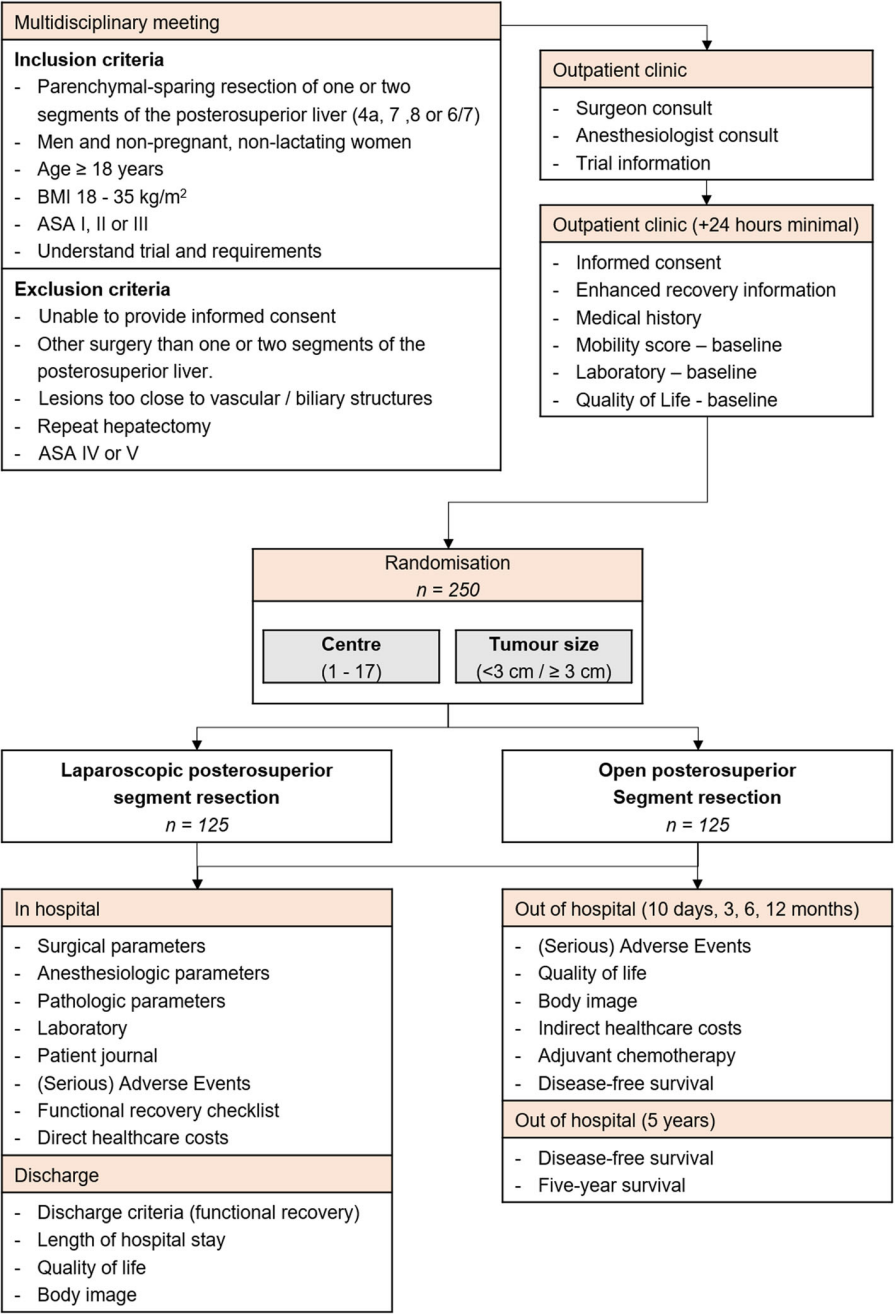
ORANGE Segments trial flow diagram

Patients are considered for a parenchymal-sparing liver resection (including wedge resections and full segmentectomies) involving one or two of segments 4a, 7, 8 or a right posterior sectionectomy (segments 6/7) only after careful evaluation by the local multidisciplinary board that includes surgeons, radiologists, pathologists, gastroenterologists, oncologists and radiotherapists. Patients who fulfil the eligibility criteria are randomly assigned to the laparoscopic or open group (Fig. 1).

#### Intervention group

Patients are treated with parenchymal-sparing posterosuperior liver segment resection using a laparoscopic approach. The surgical technique in this study is not standardised. Surgeons in the participating centres are free to use their preferred technique and surgical devices to gain intra-abdominal access, perform parenchymal transection and maintain vascular control. The ports are placed as preferred by the surgeon.

#### Control group

Patients will undergo liver resection in the same location and extent as described for the intervention group. Access to the liver is through an incision of the surgeon’s choice. There is also no standardised technique for transection, vascular control and wound closure.

### Perioperative care

Daily guidelines of the pre-, intra- and postoperative care of patients undergoing major liver resection in the enhanced recovery programme are followed as previously described [37, 38].

### Primary endpoint

**Fig. 2:**
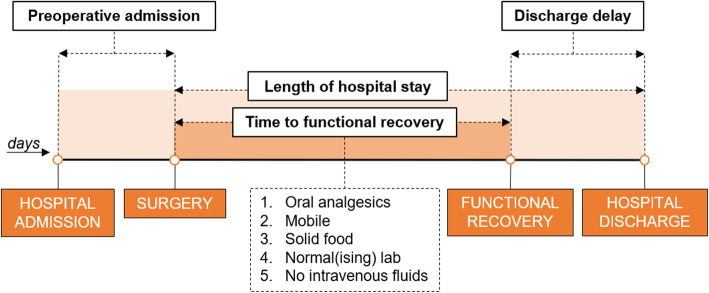
Functional recovery criteria

The primary endpoint of the trial is time to functional recovery. A patient is considered functionally recovered if he or she:

- Has adequate pain control with oral analgesics only
- Is independently mobile at the preoperative level; as objectified with a mobility score of 8 or higher (supplementary Fig. 2) [39]
- Tolerates solid food for at least 24 h
- Has a normal or decreasing total bilirubin, alanine aminotransferase and aspartate aminotransferase and a normal international normalised ratio (INR)or an INR of at least 80% of its normal value
- Is independent of intravenous fluid administration
- When all of these criteria are met, it is considered to be medically justified to discharge a patient, provided the patient is willing to go home (Fig. 2).

### Secondary endpoints

Secondary endpoints comprise short- and long-term surgical and oncological outcomes. These include intraoperative blood loss, operation time, intraoperative incidents (Satava classification), length of hospital stay, morbidity, liver-specific morbidity, 90-day mortality, readmission percentage, resection margin, quality of life, body image, incidence of incisional hernia at 1 year, hospital and societal costs for 1 year, time to adjuvant chemotherapy initiation, disease-free survival and overall survival [40–42].

### Sample size

Data on time to functional recovery in liver surgery is scarce. In the absence of such data, hospital length of stay is used instead of time to functional recovery to calculate the required sample size. In a multicentre propensity score-matched analysis of laparoscopic versus open resections of posterosuperior liver segments, the median hospital stay was 4 days (range 1–11 days) for the patients undergoing laparoscopic liver resections compared to 6 days (range 3–44 days) for the group operated by the conventional open procedure [19].

The aim of the ORANGE Segments trial is to show a reduction in time to functional recovery of 2 days in the laparoscopic group compared to the open surgery control group. This is considered to be clinically meaningful. Assuming a standard deviation of 5.0 days, the implied effect size *d* becomes 0.40. Taking into account a drop-out rate of 10% and the loss of some degrees of freedom for covariate effect estimation (centre and tumour size), a total sample size of 250 patients (125 per group) is planned to demonstrate a 2-day reduction in time to functional recovery with a level of significance alpha = 0.04, two-tailed and a power of 80%. An alpha = 0.04 was used instead of 0.05 in view of a planned interim analysis halfway the trial with an alpha = 0.01 to preserve an overall type I error rate of 5% [43].

### Randomisation and blinding

Randomisations are performed using a randomisation software (ALEA®, online randomisation module), a concealed central computer-generated randomisation service, and carried out by the local principal investigator, authorised researcher or research nurse. Patients are randomised using the minimisation method to balance the two groups for tumour size (≥3 cm/ or < \3 cm) and centre [44, 45]. Normal allocation chance is set to 50% (1:1). Only after imbalance of 2 patients within a given centre and tumour size factor, the maximal probability to be assigned to the underrepresented group is set to 90%. For transparency and control, each individual treatment allocation chance is registered. Patient confidentiality is guaranteed by applying pseudo-anonymisation. The patient identification keys are stored locally by each principal investigator.

The patient, ward physicians and ward nurses are blinded to the surgical procedure by applying a large abdominal dressing to cover all surgical incisions until postoperative day 4. The operating schedules and surgical reports are also adjusted by the principal investigators to prevent any unblinding before functional recovery or postoperative day 4. The medical and nursing staff can be unblinded if the patients’ condition necessitates this. Patient blinding adequacy is assessed by asking the patient on postoperative day 2 whether open or laparoscopic surgery has been performed.

### Implementation

A member of each local research team will be responsible for the randomisation process and will pass the assigned treatment to the treating surgeon and the operating room staff. Eligible patients will be offered to participate when a resection in the posterosuperior liver segments is planned by the surgeon. The assigned treatment is carried out by the surgeon and the postoperative care is standardised according to the local enhanced recovery after surgery protocol. The surgeon plays no role in postoperative assessments.

### Follow-up

Postoperative morbidity is recorded prospectively for 90 days, classified according to the Accordion Severity Grading System of Surgical Complications and marked as adverse or serious adverse events [46, 47].

The total follow-up duration is 5 years from the date of surgery (Fig. 1). Outpatient follow-up visits take place at the discretion of the responsible physician. Ninety-day mortality and overall survival are determined respectively 3 months and 5 years postoperatively. In addition, disease recurrence is monitored at 3, 6 and 12 months after surgery. If applicable, the use of preoperative chemotherapy and the time to start adjuvant chemotherapy are registered preoperatively and at 3, 6 and 12 months postoperatively.

Surveys are carried out as a telephone interview, via mail or online (Fig. 1). The EuroQol EQ-5D-3L status test and the European Organisation for Research and Treatment of Cancer QLQ-C30 with the LM21 module are used to assess patient’s quality of life [48–50]. Assessment of the patients’ quality of life is performed preoperatively, at hospital discharge and 10 days, 3, 6 and 12 months postoperatively. The Body Image Questionnaire is used to evaluate postoperative body image and cosmesis at hospital discharge and 10 days, 3, 6 and 12 months postoperatively [51, 52]. Incisional hernia incidence is assessed with CT or ultrasound examination 12 months postoperatively.

Direct liver surgery expenses consist of personnel and material costs. Total operating time, operating theatre and material costs (e.g. disposables, monitors, endoscopic tower), personnel costs (surgeon, anaesthesiologist, assistants, operating nurses) and hospital stay are documented. Unit prices are based on prices from the participating centre financial departments or, if unavailable, are derived from general national guidelines for pricing.

All costs related to readmission are added to the total hospital expenses. Furthermore, postoperative outpatient clinic visits, general practitioner consultations and home care costs are documented and quantified.

A cost questionnaire offered at 3, 6 and 12 months postoperatively assesses the societal and individual costs due to patients’ work absence and the impact of the surgery on work and normal daily activities. For patients performing paid labour, productivity loss is calculated using the human-capital approach, which counts any hour not worked as an hour lost. The incremental costs per quality-adjusted life year gained are based on utility scores from the EQ-5D-3L combined with the total (direct and indirect) patient costs [48, 49].

### Pathology

Resection margins are defined as the shortest distance of the lesion(s) to the resection plane in millimetres and classified as R0 shortest margin ≥1 mm (microscopically radical), R1 shortest margin < 1 mm (microscopically involved) and R2 no margin (macroscopically visible tumour on the cut surface).

### Baseline characteristics

Baseline criteria are ascertained on the day of admission to the hospital. These include age, sex, centre, body mass index, disease characteristics, preoperative treatment if applicable and prior abdominal surgery. Preoperatively, patient’s comorbidities are registered descriptively and summarised with the American Society of Anaesthesiologists (ASA) physical status classification system. Venous blood samples are drawn and analysed for blood group, haemoglobin, leucocytes, platelets, prothrombin time, activated partial thromboplastin time, international normalised ratio, renal function, liver, C-reactive protein and if applicable carcinoembryonic antigen and alpha-fetoprotein.

### Data collection

All patient data are prospectively collected either on paper case record forms (CRFs) or directly entered into eCRFs on Castor® (Castor EDC (2019), Castor Electronic Data Capture, Amsterdam, The Netherlands). All data are stored in compliance with good clinical practice guidelines on a European Union-based server, referring to the patient’s trial identification code (created by the randomisation software) to ensure patient confidentiality. Local researchers have access to their site data. Only the principal and coordinating investigator and the trial statistician have access to the final dataset. The trial coordinator will conduct regular audits regarding data completeness and plausibility. Data is kept for 15 years after completion of the trial.

### Statistical analysis

The primary hypothesis of the ORANGE Segments trial is that laparoscopic resection of the posterosuperior liver segments is superior to an open approach, in terms of short- and long-term surgical and oncological outcomes, when performed in an enhanced recovery setting. To assess these hypotheses, first, an intention-to-treat (ITT) approach is used: all randomised patients with available study data and who did not withdraw consent are analysed according to their treatment allocation, regardless of whether the surgical procedure was completed as scheduled. Only patients that do not receive surgery due to withdrawal by the investigator are considered a drop-out and will not be analysed in the ITT analysis. Secondary to the ITT analysis, a per-protocol analysis is done which includes all patients according to the treatment they actually received. Patients for whom the surgery was converted (i.e. changed intraoperatively from laparoscopic to hand-assisted laparoscopy or open) remain within the laparoscopic group for all analyses, as conversion is an intrinsic risk of laparoscopy. Analyses are performed in line with the CONSORT statement recommendations [53]. Baseline characteristics are presented as means and standard deviations or as medians and interquartile range, where appropriate. Standardised mean differences are explored. Time to functional recovery is measured in days and is analysed using fixed effects linear regression, considering treatment, recruiting centre, tumour size smaller/equal or larger than 3 cm, patient age, sex and tumour type as covariates at a two-tailed alpha = 0.04 (in view of the interim analysis with alpha = 0.01). Although the participating centres are not a random sample of centres in strict sense, the primary outcome analysis is repeated with centre (*n* = 17) as random instead of fixed effect as a robustness check.

All other outcomes are similarly analysed with regression (linear for quantitative outcomes, logistic for binary outcomes), applying a two-tailed alpha = 0.01 to adjust for multiple testing. Repeatedly measured outcomes are analysed with mixed regression for repeated measures to include participants with missing data. Proper and accepted methods are used for handling missing data.

Treatment by covariate interactions is explored which — if found — justify subgroup analyses. These will be presented as supplementary analyses, in view of the lower power for interaction effects and the increased risk of type I error due to multiple testing. Additional predefined exploratory subgroup analyses are performed in patients based on:

- Body mass index (normal, overweight and obesity)
- Tumour type (colorectal liver metastasis, hepatocellular carcinoma, cholangiocarcinoma and other malignant tumours)
- Preoperative systemic treatment (yes or no)
- Previous abdominal surgery (yes or no)
- World Health Organization performance status (0/1 or 2 and above)
- American Society of Anaesthesiologists physical status classification system (grade 1 or grade 2 or grade 3)
- Conversion (yes or no)
- Intraoperative blood loss (< 250 ml, 250–500 ml, 500–1000 ml or ≥ 1000 ml)
- Intraoperative inotropic medication (yes or no)
- Postoperative complications (no, Clavien-Dindo grade 1/2 or Clavien-Dindo grade 3/higher complications)
- Composite endpoint of liver-specific morbidity (yes or no)
- Comprehensive complication index (0 ≤ 20, 20 ≤ 26, 26 ≤ 40 or 40 ≤ 100)
- Quality of liver parenchyma (normal, steatosis fibrosis or severe hepatitis/cirrhosis)

### Monitoring

An independent DSMB was appointed for this trial, consisting of three members: a chair, a methodologist and a medical specialist. In a concerted effort, a DSMB–charter has been developed and all three members have signed a non-competing interest form. The main responsibility of the DSMB was to safeguard the interests of trial participants, to assess the safety and efficacy of the interventions during the trial and to monitor the overall trial conduct. The DSMB also provided independent review and approval of the revisited statistical analysis plan before any data analysis could be initiated. Data entry completeness will be monitored at regular intervals by the trial coordinators.

### Safety

All adverse events are reported to the coordinating investigators. Adverse events are regarded as serious if these lead to death, are life-threatening (e.g. intensive care admittance) and lead to an admission longer than 10 days and a readmission within 30 days after surgery or to permanent or serious disability. All serious adverse events are reported within 24 h to the coordinating investigator of the University Hospital Southampton and the Maastricht University Medical Center+ and are reported to the Dutch Medical Research and Ethics Committee.

An interim analysis of the primary outcome and mortality was performed after inclusion of 50% of the sample (*n* = 125), applying two stopping rules: stopping for significance if a significant difference was found between both trial groups with respect to the primary outcome (time to functional recovery) at a two-tailed alpha of 0.01 and stopping for safety if mortality after resection exceeded 5% in patients with normal liver function or exceeded 10% in cirrhotic patients. The DSMB members reviewed this analysis, and as the interim analysis did not meet any of the stopping rules, the trial was continued.

### Ethics and dissemination

The Sponsor has insurance, which is in accordance with the legal requirements in the Netherlands. The participating national and international centres will provide their patients with their own insurance. The insurance applies to the damage that becomes apparent during the study or within 4 years after the end of the study.

The final report will be submitted for publication in a high-quality peer-reviewed international journal and will be presented at relevant international scientific meetings. Within the trial group and considering the international publication policy guidelines of the EORTC, IMCJE and CONSORT, the authorships have been distributed according to contribution for coordinating and participating centers. The key points, infographic and links to the final report will also be disseminated via social media platforms.

## Discussion

Laparoscopic liver surgery has been widely adopted but there is a crucial need to have a robust evidence base for such changes to practice. The ORANGE Segments trial is the first multicentre randomised controlled trial designed to assess the superiority of the laparoscopic approach to resection of the posterior superior segments compared to the open approach in terms of time to functional recovery.

The international ORANGE collaborative is a European-wide collaboration of high-volume specialist centres experienced in laparoscopic liver surgery. The consortium conducted the ORANGE II (NCT00874224), and the ORANGE II Plus trial (NCT01441856) that completed recruitment, and provided herewith information about feasibility, results and limitations of randomised controlled trials in laparoscopic liver surgery [54].

Laparoscopic resections in the PSS are considered technically challenging and should be graded as a major procedure in terms of technical complexity [12–14, 55]. The difficulty lies in the exposure in a limited workspace with the surrounding diaphragm and the rib cage, the curvilinear resection surfaces, the need for a precise trocar placement, the challenges in the ultrasonographic evaluation of the cutting edges and eventually the intricacy of controlling major bleeding. Hence, before adopting the laparoscopic approach for such resections, it is recommended that a long learning curve is completed starting with minor and easier liver resections [13, 18, 56–59].

The approach to assess surgical interventions profoundly differs from other medical specialties. Trials of investigational medicinal products must pass through multiple phases before regulatory approval and widespread clinical use. By contrast, surgical techniques often evolve and may never be truly tested in a randomised controlled trial. If such trials are conducted, the results are often not fully adopted due to criticism about methodology [60]. Nonetheless, randomised controlled trials remain the most rigorous way to examine the efficacy of a treatment and are therefore needed for a broader acceptance of surgical practice among specialists, referrers, insurers and lastly jurisdiction.

One of the primary problems with the conduct of surgical trials is that at the time a newly introduced approach or technique is evaluated, the performing surgeons lack expertise in the intervention. Consequently, trial results may be compromised and misrepresented by the lack of experience rather than the efficacy of the tested technique. Another key barrier to surgical trials is poor accrual; indeed, approximately 10% of surgical trials are suspended prematurely for this reason [61]. This may be due to a low number of centres being able to participate owing to a limited number of surgeons having the relevant expertise. It may also result from a lack of equipoise as many surgeons may have already adopted the technique successfully and the public’s attitude is in favour of the new technique. A wide and easily mobilisable network of surgeons and proper timing of the trial is thus paramount for surgical trial feasibility.

The ORANGE II trial comparing laparoscopic and open resections of the left-lateral section was terminated early due to lack of equipoise resulting in all analyses being underpowered such that no conclusions can be drawn [54]. The surgeon’s preference for the laparoscopic approach was identified as the main contributor to the poor recruitment. This was a clear example of a technique being widely adopted before a trial could be successfully performed. Furthermore, this serves to highlight the issue of the amount of time needed to perform such trials once the trial design, funding, recruitment and follow-up of participants are considered.

One major difference of the ORANGE Segments trial in comparison to other surgical trials is that functional recovery, a composite measure, was chosen as its primary endpoint. Within the framework of an enhanced recovery programme, this endpoint appears superior to length of stay for multiple reasons. Firstly, it is an actual reflection of postoperative recovery and, contradictory to length of hospital stay, is less influenced by non-clinical discharge matters such as administrative issues, problems in homecare support, logistic troubles or — within an international setting — cultural and healthcare system differences. Another important reason is trial feasibility, as sample sizes based on endpoints such as morbidity, mortality or survival require a tremendous amount of participants and are therefore ethically and financially difficult to justify and conduct [41, 62].

In conclusion, the ORANGE Segments trial is a multicentre randomised controlled superiority trial comparing laparoscopic and open parenchymal-sparing resections in the posterosuperior liver segments. The trial is conducted within an enhanced recovery programme and aims to provide evidence on the effects of laparoscopic surgery on time to functional recovery as its primary outcome. Secondary endpoints include intraoperative outcomes, length of stay, resection margin, postoperative complications, 90-day mortality, time to adjuvant chemotherapy initiation, quality of life and overall survival.

### Trial status

Eight centres in the UK (University Hospital Southampton, Derriford Hospital in Plymouth; University Hospitals Birmingham, King’s College Hospital in London; Oxford University Hospitals, Manchester Royal Infirmary, Aintree University Hospital in Liverpool; and Freeman Hospital in Newcastle), four centres in Italy (San Raffaele Hospital in Milan, San Camillo Forlanini Hospital in Rome, the Foundation Poliambulanza in Brescia and the Policlinico Federico II in Naples), two centres in the Netherlands (Amsterdam University Medical Centers and Maastricht University Medical Center+), one centre in Belgium (General Hospital Groeninge in Kortrijk), one centre in Norway (University Hospital Oslo) and one centre in Russia (Moscow Clinical Scientific Centre) gained ethical approval. The trial was registered on May 9, 2017, in the ClinicalTrials.gov register under identification number NCT03270917. The first patient was randomised on November 7, 2017. This trial is actively recruiting and has currently enrolled 219 patients.

## Supplementary Information

**Additional file 1: Supplementary Fig. 1.** SPIRIT figure [36].

**Additional file 2: Supplementary Fig. 2.** Mobility score. The mobility score has been adapted from the Groningen Activity Restriction Scale for Measuring Disability [39].

**Additional file 3: Supplementary document 1.** SPIRIT checklist.

## Data Availability

All data generated or analysed during this study are included in the published results. The source data are kept on Castor® for 15 years. The datasets analysed during the current study are available from the corresponding author upon request.

## Abbreviations

ASA: American Society of Anaesthesiology
CRF: Case record form
DSMB: Data and Safety Monitoring Board
EDC: Electronic data capture
GCP: Good clinical practice
INR: International normalised ratio
ITT: Intention-to-treat
ORANGE: Optimised recovery with accelerated nutrition and gastro-intestinal enhancement
PSS: Posterosuperior segments
RCT: Randomised controlled trial
