# Supplementary figures and images for: Laparoscopic versus open resections in the posterosuperior liver segments within an enhanced recovery programme (ORANGE Segments): study protocol for a multicentre randomised controlled trial
Source: Trials. 2022 Mar 9;23:206. doi: 10.1186/s13063-022-06112-3 (PMC8908665; doi:10.1186/s13063-022-06112-3)

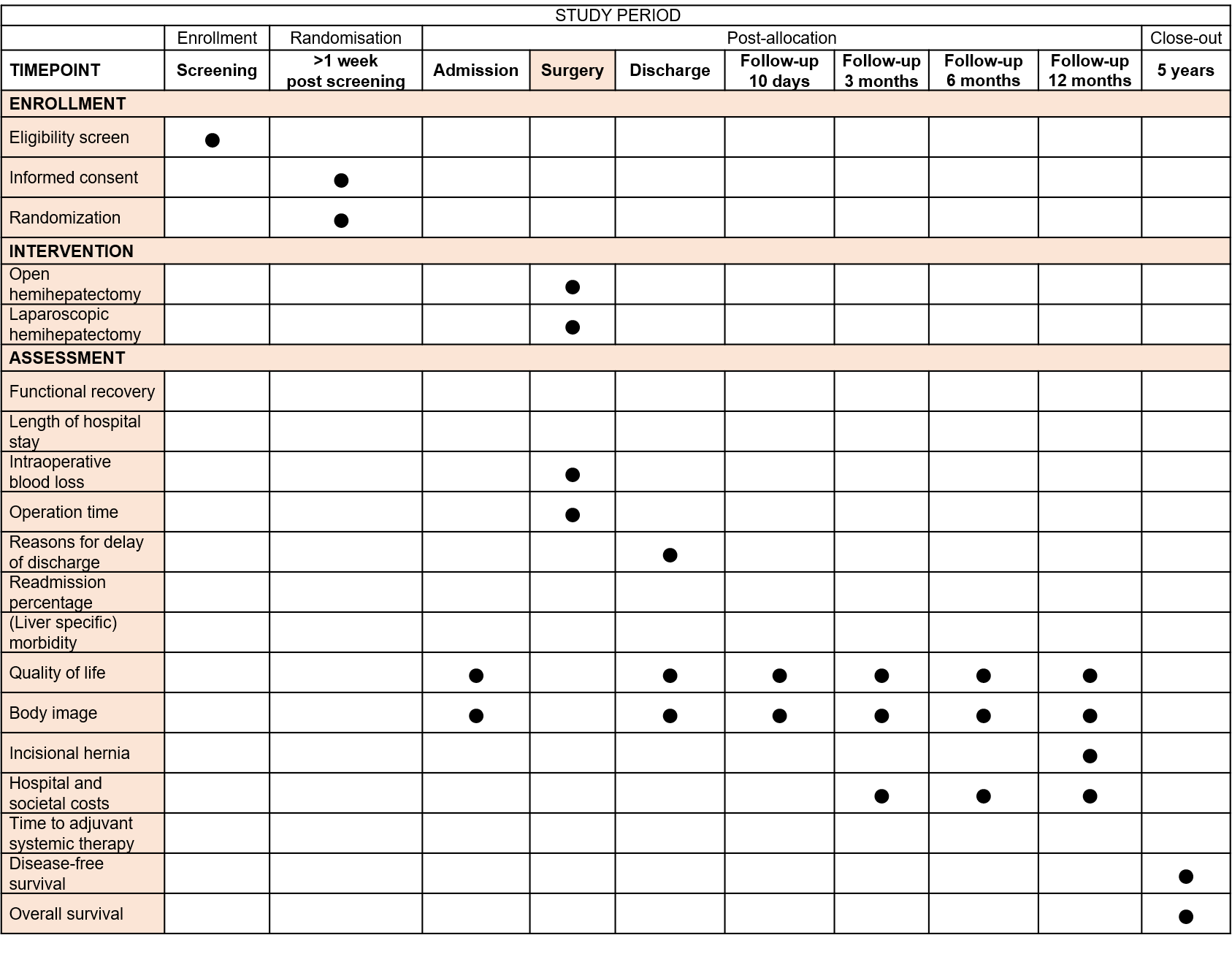

Supplement: Supplementary file 1 — Additional file 1: Supplementary Fig. 1. SPIRIT figure [36]. [file 13063_2022_6112_MOESM1_ESM.tif]

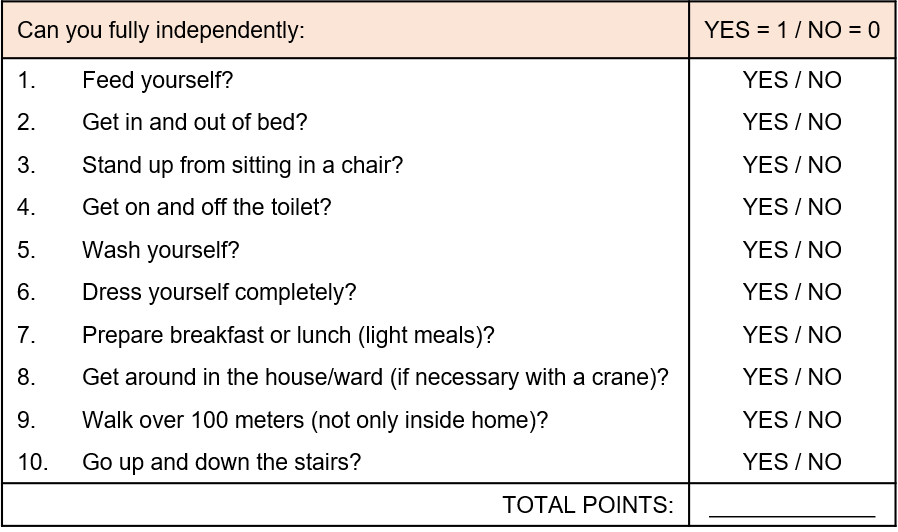

Supplement: Supplementary file 2 — Additional file 2: Supplementary Fig. 2. Mobility score. The mobility score has been adapted from the Groningen Activity Restriction Scale for Measuring Disability [39]. [file 13063_2022_6112_MOESM2_ESM.tif]
